# Transcriptome variation in human populations and its potential application in forensics

**DOI:** 10.1007/s13353-019-00510-1

**Published:** 2019-08-10

**Authors:** P. Daca-Roszak, E. Zietkiewicz

**Affiliations:** Institute of Human Genetics, Polish Academy of Sciences, Strzeszynska 32, 60-479 Poznan, Poland

**Keywords:** Transcriptome variation, Human population identification, Laser capture microdissection, Forensic identification

## Abstract

This review presents the state-of-the-art in the forensic application of genetic methods driven by the research in population transcriptomics. In the first part of the review, the constraints of using classical genomic markers are shortly reviewed. In the second part, the developments in the field of inter-population diversity at the transcriptomic level are presented. Subsequently, a potential of population-specific transcriptomic markers in forensic science applications, including ascertaining population affiliation of human samples and cell mixtures separation, are presented.

## Genetics in forensic identification

Genetics has been long recognized and adopted as an efficient and reliable approach to forensic identification (FID) of human samples. Genetic-based FID may be perceived from different perspectives, depending on the goal of the investigation. These goals vary considerably and may concern:

- determination of family links
- identification of individuals from whom forensic biological traces derive
- assessment of the ancestral contribution and of individual’s affiliation with continental/ethnic groups
- finding clues about the inherited or acquired phenotype
- identification of the tissue source of a biological material

**Fig. 1:**
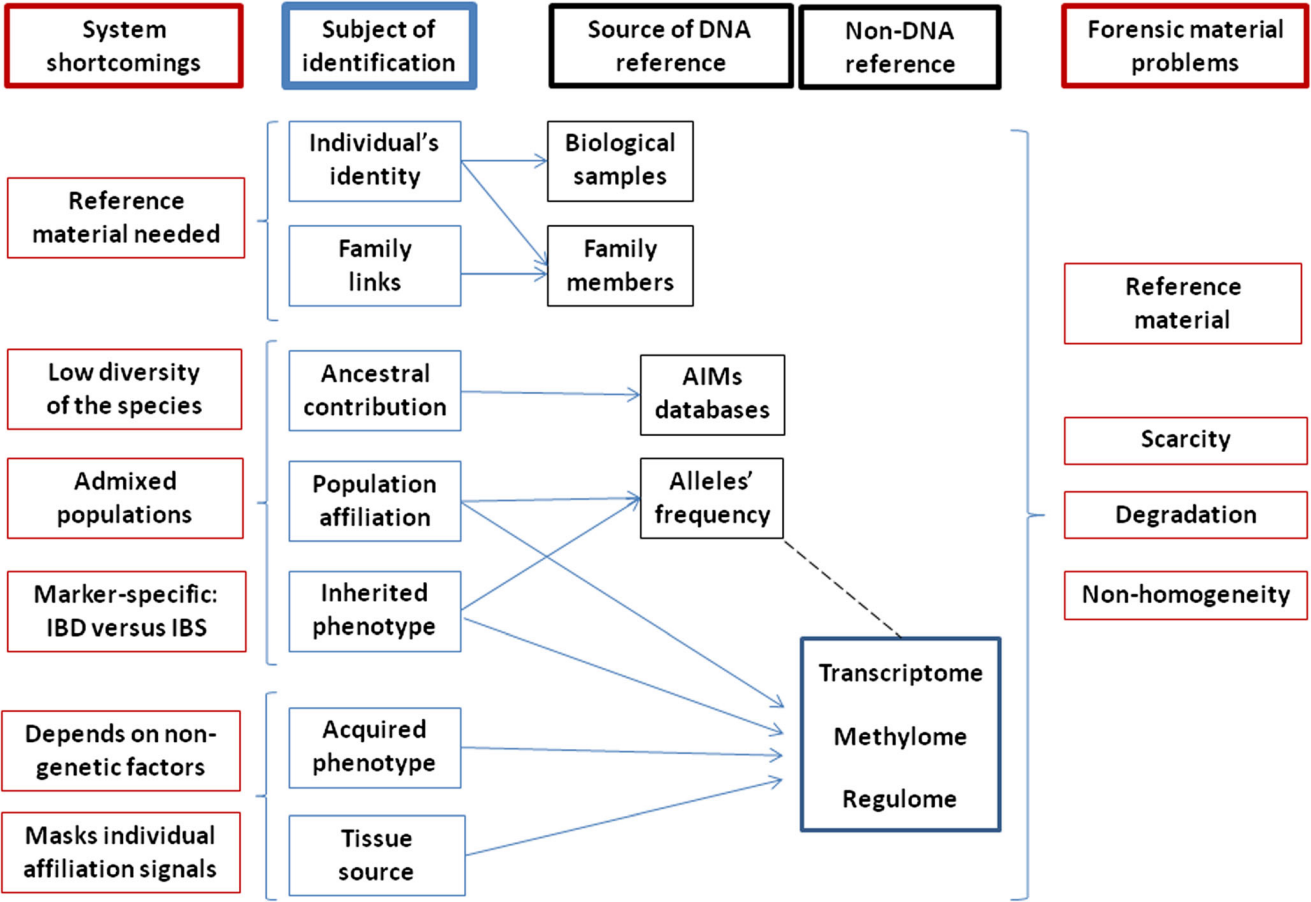
Forensic identification—schematic representation of the relation between forensic goals and analytical approaches. The different entries in the “subject of identification” column are interdependent—e.g., the information of the population affiliation or the phenotype of the forensic sample may support the proper identification of the individual

Each of the goals in FID requires using appropriate genetic markers and specific methodology, to counteract numerous and variable constrains associated with the analysis (See Fig. 1). Besides the intrinsic constraints associated with the limited information of genetic markers, there are practical concerns in genetic FID, related to any of the following: lack of reference samples, scarcity and/or degradation of the genetic material in forensic traces, and non-homogeneous character of the material (mixed samples).

There is ample literature describing the application of classical, DNA-based genetic markers (microsatellites, SNPs, and haplotypes) for resolving family links (paternity, family relations) and individual’s identity as compared with the reference (for the review, see for example Zietkiewicz et al. 2012).

Considerable progress has also been achieved in using DNA markers to assess ancestral contribution to the genome make-up of individuals and thus population affiliation of unknown samples (e.g., Elhaik et al. 2014; Santos et al. 2016). Rapid development of the analysis of human transcriptome and epigenome variability has opened further perspectives, both in the context of tissue identification, and determination of phenotypes; these aspects have been extensively applied in forensic studies (e.g., Frumkin et al. 2011; Xu et al. 2014; Kader and Ghai 2015; Park et al. 2016; Zubakov et al. 2016). Importantly, both transcriptomic and epigenomics markers may become useful in those of FID endeavors, which require determination of population affinity of the forensic material.

The aim of this review is to present the state-of-the art in the forensic application of genetic methods driven by the research in population genomics and transcriptomics, in the context of some of the major FID problems: the lack of reference samples and non-homogeneity of the biological material.

## Genome diversity of human population in FID

The majority of genetic methods used in FID rely on the comparison of a material under investigation with reference samples (from suspected individuals, forensic archives, family members, etc.); sometimes, the information stored in a variety of specialized genetic databases can be used. However, the reference data are often unavailable to an investigator. In such cases, an alternative tactic may be applied: to assign an unidentified biological sample to a specific human population by comparing it with population-specific data. While indirect, it allows narrowing the focus of the investigation. Consequently, ascertaining population/ethnic affiliation of human samples based on DNA profiling has recently become an important goal in many forensic fields: e.g., crime perpetrator detection, identification of mass disasters or terrorist attack victims (Zietkiewicz et al. 2012; Chakraborty et al. 1999; Budowle et al. 2005; Phillips et al. 2009; Mamedov et al. 2010; Bamshad et al. 2003). The basic shortcoming of population-differentiating genomic markers is related to the low diversity of human species: the majority of genetic variance is shared by all human groups, reflecting the relatively young evolutionary age of our species and/or the recent gene flow (admixture) among extant populations (e.g., Shriver 1997; Zietkiewicz and Labuda 2001; Tishkoff and Williams 2002). In consequence, what actually differentiate human populations are different allele frequencies rather than the presence or absence of marker alleles. Markers with significant frequency differences between human populations are often referred as ancestry informative markers (AIMs) (Frudakis et al. 2003; Shriver and Kittles 2004; Nassir et al. 2009). Relatively few AIMs are needed for differentiating populations that have diverged a long time ago (e.g., continental groups). However, in case of closely related populations, which share very recent evolutionary history, the sufficient discrimination power can be achieved only by analyzing a very large number of markers distributed over the whole genome (Lao et al. 2006). These analyses usually rely on microarray-based technology (e.g., Novembre et al. 2008; Barbosa et al. 2017).

The majority of population-differentiating markers are selectively neutral, and they mostly reflect the demographic history that shaped the present-day population diversity. AIMs used to infer the ethnic origin of individuals are usually selected from a variety of genomic SNPs, SNP-based haplotypes or CNVs (copy number variants); they may be diploid or haploid (mtDNA, Y-chromosome). Of note, fast-mutating microsatellites (simple tandem repeats (STRs)), which are the most informative markers for the identification of individuals compared with the reference samples, are rarely used as AIMs. This is due to the fact that distinguishing alleles identical by descent (IBD) from those identical by state (IBS) is a challenging task, and conclusions on the population affiliation or the ancestry of the sample are not straightforward.

Many effective population-specific tests have been designed based on markers linked with the genes subjected to selection, e.g., involved in the metabolism of xenobiotics, immune response, fertility, or pigmentation (e.g., Phillips et al. 2007; Rogalla et al. 2015). While these markers can be successfully used to differentiate populations, it has to be remembered that some of the allele frequency similarities, rather than reflecting common ancestry, could be a result of polyphyletic mechanisms that depend on the environment and act in multiple populations independently.

The aforementioned constraints seriously limit the efficiency of DNA-based markers in applications, which require population-based discrimination of the biological material. Recently, intense efforts have been directed to search for non-DNA markers that would exhibit population specificity.

## Transcriptome variation among populations

The application of expression microarrays (from Affimetrix or Illumina) targeting thousands of gene transcripts has allowed exploration of the transcriptional variation in humans at the unprecedented scale. First, the levels of gene expression have been shown to differ not only among cells/tissue types, but also among individuals (Cheung et al. 2003; Monks et al. 2004; Morley et al. 2004; Stranger and Dermitzakis 2005). Soon, numerous studies have demonstrated that, while the bulk of variation in the expression level is observed between individuals, significant differences across continental populations also exist (Spielman et al. 2007; Stranger et al. 2007; Storey et al. 2007; Price et al. 2008; Zhang et al. 2008; Ye et al. 2014; Armengol et al. 2009; Fan et al. 2009; Lappalainen et al. 2013; Yin et al. 2014; Mele et al. 2015; Dimas et al. 2009; Li et al. 2014a).

### LCL-based studies

The majority of data supporting the notion of the inter-population differences in gene expression are based on the model of lymphoblastoid cell lines (LCLs) (EBV-immortalized human B-lymphocytes). The majority of LCLs, commercially available from Coriell depository and previously used in the International HapMap Project, represent ethnically homogeneous continental populations: CEU—Utah individuals of European ancestry, CHB—Han Chinese, JPT—Japanese, YRI—Nigerians,, and AA—admixed African Americans. In spite of the common source of the cell lines used in human transcriptome diversity studies, the direct comparison of the results is difficult for several reasons. First, not all the studies compared the same populations; most often, only limited pairwise comparisons were performed (CEU-CHB; CEU-YRI; YRI-CHB, etc.), and the numbers of individuals representing the populations differed. Second, different methodologies have been applied to determine the expression level (various microarray platforms from Affymetrix and Illumina, or next-generation sequencing (NGS)). Third, estimating and reporting the significance of the results was not uniform (e.g., different statistical models were used; the fold-difference was not always reported; not all the studies provided the names of best-differentiating genes; etc.).

In the seminal study of Spielman, Affymetrix HG-Focus microarray addressing over 4000 genes expressed in lymphobastoid cells was used to compare expression in Europeans (60 CEU) and Asians (41 CHB and 41 JPT) (Spielman et al. 2007). Over 1000 genes (25%) were found to be differentially expressed between Europeans and Asians (*t* test, *p* < 0.05), while only 27 genes differentiated Chinese and Japanese. Among 35 genes displaying at least 2-fold expression difference between Europeans and Asians, the best were: *UGT2B17* and *ROBO1* (with 22- and 4-fold higher expression in CEU, respectively) and *CLECSF2* (with 4-fold higher expression in YRI).

In another study using Affymetrix microarray (addressing 5190 genes expressed in LCLs), expression levels in Europeans and Africans (16 CEU and YRI) were compared using models accounting for differences between individuals as well as populations (Storey et al. 2007). Approximately 17% of the genes were differentially expressed in the two populations; the differences in 50 genes were significant at FDR < 20%, with the average fold-change of 1.65. Many of the differentially expressed genes were associated with the immunological response (e.g., gene-encoding cytokines and chemokine receptors: *CCL22*, *CCL5*, *CCR2*, *CXCR3*).

The levels of expression in Europeans and Africans were further analyzed in a larger sample of LCLs (30 CEU and 30 YRI family trios) using Affymetrix Human Exome array (over 9100 transcript clusters) and two independent statistical approaches taking into account the presence of SNPs in the probes (Zhang et al. 2008). About 4.2% of the transcript clusters displayed significantly different expression between the CEU and YRI (247 and 136 with higher expression in YRI and CEU, respectively), with the average fold-change of 1.3. Biological processes found to be enriched in the differential transcripts included ribosome biogenesis and antimicrobial humoral response, as well as cell-cell adhesion, mRNA catabolism, and tRNA processing. Nine of the genes (*DPYSL2*, *CTTN*, *PLCG1*, *SS18*, *SH2B3*, *CPNE9*, *CMAH*, *CXCR3*, and *MRPS7*) were earlier reported among the top 50 genes differentially expressed in CEU and YRI (Storey et al. 2007).

The impact of SNPs and CNV on transcriptome variation has been extensively studied using Illumina whole-genome array in 270 LCLs from CEU, CHB, JPT, and YRI populations (Stranger et al. 2007). Over 5300 genes exceeded the threshold of 16% difference in the median expression in one or more of the population pairs; assuming about 12,000 genes expressed in LCLs, the fraction of genes with significant expression differences between any two populations was estimated between 17 and 29%.

In another study, variation in gene expression was explored in 210 LCLs from four ethnic groups (CEU, CHB, JAP, and AFR), using Illumina microarray addressing more than 11,000 transcripts (Fan et al. 2009). Expression of 427 genes was characterized by higher inter-ethnic than inter-individual variance. Ten of these genes were characterized by the overall variance in expression > 8% (*CXXC4*, *KIF21A*, *LOC376138*, *RGS20*, *TBC1D4*, *TUBB*, *UGT2B11*, *UGT2B17*, *UGT2B7*, and *UTS2*); two of these genes (*UGT2B17*, *RGS20*) have been earlier reported as differentially expressed in Asians and Europeans (Spielman et al. 2007).

After the initial studies based on microarray analysis of transcriptome variability, the dynamic development of high-throughput NGS techniques resulted in a number of studies that analyzed even more transcripts. Besides confirming population differences in the level of expression of a considerable number of genes, they also shed more light upon the mechanisms underlying these differences.

In one of the NGS-based studies (Lappalainen et al. 2013) transcriptomic variation was characterized in over 460 LCLs from Africans (YRI) and four European subpopulations (CEU, FIN, GBR, and TSI). The inter-population differences accounted for only a small fraction (3%) of the total variation in expression. In spite of this, the number of genes displaying significant expression differences between Africans and Europeans was impressively high, ranging from ~ 1300 to 4300 (depending on which European subpopulation was compared with YRI). The much lower number of differentially expressed genes was seen when European subpopulations were compared.

In another NGS-based study, expression was examined in 20 LCLs from CEU and CHB (Li et al. 2014a). Over 400 differentially expressed genes were identified (including 132 and 291 with higher and lower expression in CHB, respectively); the magnitude of expression differences was modest (with the median of 2 and 0.4 for the genes up- and downregulated in CHB, respectively). Interestingly, new ethnic-specific isoforms of the known transcripts were revealed in over 200 genes (199 in CHB and 28 in CEU); eleven of those were found in the genes characterized by differential expression in the examined populations (*CLEC2B*, *ARL4C*, *ZBP1*, *ITM2B*, *c11orf21*, *UTS2*, *VCAN*, *CACNA1E*, *EFNA5*, *NR2F2*, and *MGLL*). Ethnic-specific splice junctions were found in only eight genes (*NASP*, *MTIF3*, *CCDC47*, and *TBCA* in CHB and *ITGB7*, *CRTAP, ERO1LB*, and *NSUN2* in CEU) (Li et al. 2014a).

In an RNA-sequencing analysis of 45 LCLs from seven non-European populations (Namibian San, Mbuti Pygmies, Algerian Mozabites, Pakistan, East Asia, Siberia, Mexico), 44 differently expressed genes were identified, the vast majority representing immunity pathways. The highest inter-population gene expression variation was obtained for *THNSL2*, *DRP2*, *VAV3*, *IQUB*, *BC038731*, *RAVER2*, *SYT2*, *LOC100129055*, *AK126080*, and *TTN* genes (Martin et al. 2014).

The inter-population differences in the expression level have been repeatedly shown to be heritable and linked to the variation across the human genome. Potential mechanisms include INDELs or copy number variations (CNV) (Spielman et al. 2007; Armengol et al. 2009), SNPs (e.g., Stranger et al. 2007;Storey et al. 2007; Zhang et al. 2008) or alternative splicing (Zhang et al. 2008; Lappalainen et al. 2013; Li et al. 2014a).

Genetic variants in the cis- or trans-acting regulatory elements that affect transcript abundance can be mapped as expression quantitative trait loci (eQTLs). The inter-population variation in these genes’ expression are often associated with the population differences in the allele frequencies at eQTLs (Albert and Kruglyak 2015; Kelly et al. 2017; Park et al. 2018). For example, the spectacular difference of *UGT2B17* expression difference between Asians and Europeans was shown to be associated with the higher frequency of the gene deletion in Asians (Spielman et al. 2007). The differential expression of *UGT2B17* locus among populations has also been demonstrated in the study aiming to characterize population differences in the copy number variation (CNV) (Armengol et al. 2009).

### Non-LCL studies

All the examples discussed above concerned population differences in gene expression studied in LCLs. In the last few years, several studies have demonstrated that population differences, similar to those reported in LCLs, are also observed in the cell types other than immortalized B lymphocytes.

In one of these studies, population patterns of gene expression were examined in epidermal samples from 30 individuals representing three continental populations (Yin et al. 2014). Microarray analysis has revealed 14 genes with more than 1.5-fold expression differences between Africans, Caucasians, and Asians. Not surprisingly, the strongest effect was seen between Africans and non-Africans, with 15- and 9-fold differences in the transcription of two best-discriminating genes (*CCL18* and *ADRA2C*, respectively). The differences between Caucasians and Asians were less pronounced, with only one gene, *NINL*, displaying a 3-fold difference in the expression level (Altshuler et al. 2012; Yin et al. 2014).

In another study, focused on population differences in the transcriptional responses of the CD4+ T lymphocytes to the conditions that mimic activation through the antigen-specific receptors (Ye et al. 2014), the set of 236-transcripts was analyzed by Nanostring profiling in 348 donors of African, European, and Asian origin. A trend towards the higher T cell activation in donors of African ancestry has been found to be associated with population differences in the mean expression of a number of genes. For example, expression of *IL2RA* (cytokine receptor) in activated T cells from Africans was approximately 15% higher than in Europeans; other differentially responsive genes included *IL17* family cytokines (over-induced in Africans) and *IFNG* (over-induced in non-Africans).

To exclude the influence of environmental factors (e.g., donor age, time of sampling) on gene expression patterns, the inter-population gene expression variation in placenta was examined in samples from four human populations: African Americans, European-Americans, South Asian Americans, and East Asian Americans (Hughes et al. 2015). The analyses revealed approximately 8% of variation in gene expression among the studied groups. African and South Asian populations had the highest inter-population variation in gene expression (> 140 genes). Genes characterized by the highest inter-population variation were mainly involved in pathways related to immune response, cell signaling, and metabolism (Hughes et al. 2015).

In the recent study on the multi-tissue transcriptomic patterns in Caucasians and Africans, population differences in the expression of over 220 protein-coding genes and 150 lncRNAs (long non-coding RNA) were reported (Mele et al. 2015). However, some of these differentiating markers were specific to individual tissue types. This is consistent with the earlier study, where the direct comparison of gene expression profiles in three types of cells (LCLs, T cells, and primary fibroblasts) has revealed that the majority (80–90%) of genomic variants affecting gene regulation act in a cell type–specific manner (Dimas et al. 2009).

The latter studies have indicated that further surveys are needed to elucidate whether any of the reported population differences in gene expression is common to different cell types. Expression profiling aiming to distinguish ethnic affiliation of the forensic samples would therefore require that the levels of transcripts are compared in the corresponding tissues.

Tissue Expression project (GTEX) may overcome the scarcity of expression data from different human tissues, other than LCLs. GTEX catalogs gene expression variation in major tissues and, additionally, provides an information regarding genetic background underlying this variation (Lonsdale et al. 2013). So far, gene expression profiles for more than 50 human tissues have been cataloged and made publicly available in GTEX database. Hitherto, GTEX project encompasses only data gathered from the Caucasian cohort, which limits applicability of the data to the global population context (Lonsdale et al. 2013).

The application of NGS in human population studies, besides revealing differences in gene expression patterns between distinct human groups (discussed above), provided the knowledge about the diversity of mRNA isoforms in human populations (Park et al. 2018; Djebali et al. 2012; Vaquero-Garcia et al. 2016). It is well known that the vast majority of human genes are subjected to alternative splicing, and a number of isoforms from a single gene may be generated (Pan et al. 2008; Wang et al. 2008; Djebali et al. 2012; Vaquero-Garcia et al. 2016; Park et al. 2018). Various mRNA isoforms have distinct stability and biological function. All these differences in the quantitative and qualitative composition of mRNA isoforms may be adopted as potential population markers.

The latest achievements in the research on alternative splicing variation in human populations have been summarized in the review by Park et al. (2018). The landscape of alternative splicing in relation to the genetic variation has been investigated in a few studies (e.g., Martin et al. 2014; Lappalainen et al. 2013; Battle et al. 2014). Most of the studies were conducted on LCL samples, and they concentrated on the mechanisms underlying formation of RNA isoforms (e.g., Montgomery et al. 2010; Pickrell et al. 2010).

For example, over 170 genes with transcript isoforms changes were identified in the European population (Kwan et al. 2008). Another study has shown that the majority (75 ± 22%) of population-specific variance in gene expression levels observed among seven global human populations can be explained by the variation in gene expression, while only the minor part is caused by the alternative splicing (Martin et al. 2014). This observation was also confirmed in the study of lymphoblastic cells from 69 Yoruban and 60 Caucasian individuals, where RNA sequencing identified 44 genes, for which the ratios of splicing isoforms were similar within each population but more different when comparing populations (Gonzalez-Porta et al. 2012).

The literature data presented above clearly indicate that a significant variation in the expression level across populations exists and that it is at least partially caused by the genomic variation.

The use of specific mRNA transcripts allows efficient differentiation of samples that originate from human populations. Our recent study has revealed two such population-discriminating transcripts: *UTS2* and *UGT2B17* (Daca-Roszak et al. 2018). These mRNA markers exhibited significant population differences in the expression level in both B cell lines and in the peripheral blood and enabled differentiation of Caucasian and Chinese cohorts with high specificity (> 90%) and sensitivity (> 76%) (Daca-Roszak et al. 2018).

## Population-specific transcriptome variation—prospects for FID applications

The aforementioned data indicate that carefully chosen population-specific transcriptomic markers can be used in FID applications in a similar way to the DNA-based AIMs, to indicate the population origin of a forensic sample. On the other hand, transcriptomic markers are, just like population-specific DNA markers, more quantitative than qualitative. Moreover, they are expected to be even more susceptible to environmental influences (age, diet). On the other hand, as discussed below, population-specific transcriptomic markers harbor an important, new potential, which may be prospectively used to solve the great problem of FID application, that of sample mixtures.

The effective use of population-specific genetic markers in FID is often hampered by the non-homogeneity of a forensic material. While deconvolution of allelic profiles obtained from mixed samples is possible (e.g., Hu et al. 2014; van der Gaag et al. 2016), it remains a difficult task and often requires using sophisticated mathematical models (e.g., Bille et al. 2014; Bieber et al. 2016). In practice, identification of multiple contributors by genotyping DNA markers in forensic samples is challenging or not feasible if the reference DNA profiles are not available (Fregeau et al. 2003; Westen et al. 2009). All these features limit the direct use of genetic markers for the analysis of evidentiary samples, which often contain mixed genetic material of unknown origin. Mixtures of cells from the same tissue type, originating from different individuals, are often encountered in the forensic evidence; in the absence of the reference samples, distinguishing the population origin of the individuals, who are the source of such material, poses a serious problem in the FID practice (e.g., Bieber et al. 2016; Gill et al. 2006).

Physical separation of DNA mixtures can be used to address complex DNA mixture problem. In fact various single cell separation technologies have been used before, mainly in sexual offense cases (e.g., Li et al. 2014b; Williamson et al. 2018) and other examples of tissue separation. However, such idea is brand new in a population-discrimination aspect.

A new perspective is related to a potential application of transcripts characterized by population-specific differences in the expression level. The idea relies on the combination of two techniques: labeling transcripts with population-specific probes and separation of the labeled cells.

**Fig. 2:**
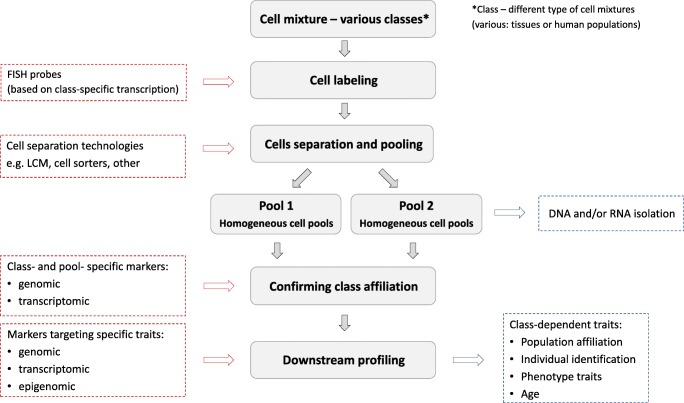
Potential application of transcriptome variation for mixture deconvolution with the use of LCM technology and FISH labeling

The cells from donors of different ethnic background could be “barcoded” with the FISH probes that specifically hybridize to transcripts characterized by differential expression in the relevant populations. In the next step, specifically labeled cells could be separated based on using the cell sorters, laser capture microdissection (LCM) technology, or any other cell separation technique (e.g., Fend et al. 1999; Datta et al. 2015) (see Fig. 2).

Population affiliation of the separated cell pools can be then confirmed by using markers appropriate for the analyzed populations. Markers may be chosen from among transcriptomic probes or genomic eQTLs (SNPs or INDELs) that underlie or associate with the differential expression; population-specific genomic markers not associated with the expression differences (e.g., Daca-Roszak et al. 2016) could be also used for this purpose. The homogenized cell pools can be further used for the downstream profiling using individual-specific or phenotype-specific markers (Vidaki et al. 2013; Zubakov et al. 2010; Zbiec-Piekarska et al. 2015; Koch and Wagner 2011; Bocklandt et al. 2011; Hannum et al. 2013; Weidner et al. 2014).

In most of the forensic cases, DNA/RNA co-isolation from the biological material is possible. The feasibility of combined DNA and RNA profiling of body fluids and contact traces, providing information about both the cell type and sample donor identity, has been reported (Lindenbergh et al. 2012). One can envision that the simultaneous analysis of two transcriptomic markers, one differentiating populations and the other—tissues, combined with any cell separation technique, e.g., LCM technology, could be the way to examine forensic cell mixtures.

Prospects of combining application of distinct markers (transcriptomic, genomic, and epigenetic) for FID purposes, however tempting, are not without flaws. From a technical point of view, the application of transcriptional probes in LCM-based separation of forensic mixtures is, at the present moment, time-consuming and expensive and requires highly qualified and experienced staff. When the amount of a material in the mixed sample is large enough, LCM can be replaced by cell sorters; however, the cell-sorter technology is predictably less suitable for the forensic purposes, where typically only a scarce amount of the evidentiary material is available.

So far, the use of population-specific transcriptomic markers and probes has not been tested in practical forensic applications. The majority of studies were performed in LCLs, cultured under specific laboratory conditions and derived from a limited set of, mostly continental, populations (e.g., Spielman et al. 2007; Stranger et al. 2007; Storey et al. 2007). Further studies are therefore required to assess sensitivity, specificity, and stability of population-specific transcriptomic markers in real-life samples, which may contain different cell types, like full blood, epithelium, sperm, and hair. Furthermore, additional search for transcripts differentiating more closely related and/or admixed human groups have to be performed. Other problems are related to the non-uniform biological basis of the transcriptome variance (e.g., some transcripts’ levels depend on the environmental factors). Therefore, when selecting transcriptomic markers to be used in the assessment of population affiliation, it will be important to exclude the genes whose expression is known to depend on gender and environmental conditions (diet, stress, etc.).

All the limitation notwithstanding, further exploration of the population-specific transcriptome variation should be the goal of research aiming to improve the applicative prospects in the field of forensic identification.

## Abbreviations

FID: Forensic identification
AIM: Ancestry Informative marker
LCLs: Lymphoblastoid cell lines
EBV: Epstein-Barr virus
IBD: Markers identical by identical by descent
IBS: Markers identical by state
LCM: Laser capture microdissection
